# Pancreatic Cancer detection via Galectin-1-targeted Thermoacoustic Imaging: validation in an *in vivo* heterozygosity model

**DOI:** 10.7150/thno.45994

**Published:** 2020-07-14

**Authors:** Huan Qin, Baohua Qin, Chang Yuan, Qun Chen, Da Xing

**Affiliations:** 1 MOE Key Laboratory of Laser Life Science & Institute of Laser Life Science, College of Biophotonics, South China Normal University, Guangzhou 510631, China.; 2 Guangdong Provincial Key Laboratory of Laser Life Science, College of Biophotonics, South China Normal University, Guangzhou 510631, China.

**Keywords:** thermoacoustic imaging, small pancreatic tumors, nanoparticles

## Abstract

**Purpose:** To investigate the feasibility of microwave-induced thermoacoustic imaging (MTAI) in detecting small pancreatic tumors (< 10 mm in diameter) and to complement the limitation of current clinical imaging methods.

**Methods:** A home-made MTAI system composed of a portable antenna and pulsed microwave generator was developed. The thermoacoustic nanoparticles were composed of the galectin-1 antibody for targeting pancreatic tumors and Fe_3_O_4_ nanoparticles as microwave absorbers (anti-Gal1-Fe_3_O_4_ nanoparticles). The microwave absorption properties of the nanoparticles were measured with a vector network analyzer and the resolving power of MTAI was investigated by imaging excised pancreatic tumors of different sizes (diameters of 1.0 mm, 3.1 mm, 5.0 mm, 7.2 mm). To simulate actual imaging scenarios, an *in vivo* heterozygosity model was constructed by covering the pancreatic tumors (~ 3 mm in diameter) in BALB/c nude mice with biologic tissue (~ 5 cm in depth). MTAI images of the heterozygosity model were acquired with/without the injection of the anti-Gal1-Fe_3_O_4_ nanoparticles and the thermoacoustic contrast from pancreatic tumors was evaluated with Student's paired t test. The data were analyzed with analysis of variance and nonparametric statistics.

**Results**: Following intravenous infusion, anti-Gal1-Fe_3_O_4_ nanoparticles efficiently accumulated in the tumor. The MTAI contrast enhancement in pancreatic tumors with anti-Gal1-Fe_3_O_4_ nanoparticles was verified *in vitro* and *in vivo*. The pancreatic tumors were visible in nude mice examined with MTAI with a mean contrast enhancement ratio of 2.3 ± 0.15 (standard error of the mean) (*P* =. 001) at 6 h post-injection of the nanoparticles. MTAI identified tiny pancreatic tumors in deep tissues with high fidelity.

**Conclusion**: MTAI offers deep imaging depth and high contrast when used with anti-Gal1-Fe_3_O_4_ nanoparticles. It can identify pancreatic tumors smaller than 5 mm, which is beyond the identification limit size (~10 mm) of other nondestructive clinical imaging methods. Thus, MTAI has great potential as an alternative imaging modality for early pancreatic cancer detection.

## Introduction

Pancreatic cancer is one of the most lethal malignancies in humans with a five-year survival rate of less than 5% 1-2. Earlier tumor detection can improve the 5-year survival rate—particularly in subjects with tumors smaller than 10 mm in diameter 1-3. Current clinical imaging methods, such as ultrasound imaging (USI), computed tomography (CT), and magnetic resonance imaging (MRI), each has advantages and disadvantages in pancreatic cancer detection.

USI is simple, economical, non-invasive, and repeatable, but it is of limited use in detecting pancreatic tumors smaller than 10 mm in diameter due to the small difference in acoustic impedance between tumors and background tissues 4. CT does not identify tumors in 33% of pancreatic tumors <15 mm in diameter 5. This low sensitivity for detecting tumors may be due to the size and the pathological characteristics of the tumor: lower tumor cellularity, more frequent intratumoral acinar tissues and islet cells, and less prominent tumor necrosis 6. MRI can detect solid pancreatic neoplasms with extremely dense cellularity or extracellular fibrosis, especially when combined with a low apparent diffusion coefficient (ADC) in the tumor 7. However, MRI is limited in definitively discriminating inflammatory and neoplastic solid lesions because of an overlap in ADC values between these two types 7. Multi detector computed tomography (MDCT) scan combined with a contrast medium injection protocol avoids missing parts of the target, but this conservative approach could result in an undesired increase of the radiation dose to the normal pancreatic tissues surrounding the lesion 8. Gadolinium-enhanced MR imaging has been shown to be equivalent or even superior to helical CT for imaging the pancreas 9. Mangafodipir trisodium-enhanced MR imaging offers advantages in the detection of small pancreatic malignancies and of liver metastases but this technique and helical CT are equivocal for local staging of pancreatic cancer 10.

MTAI is a promising new imaging modality, combining the high contrast of microwaves and the high resolution of ultrasound. Thermoacoustic imaging (TA) signal generation is a result of microwave-induced thermal effects 11. Small temperature rise can be produced when biological tissue is irradiated by a microwave pulse of adequate energy. The heated structure then thermally expands and contracts, thus producing a source of an acoustic pressure wave. MTAI detects the sound waves and reconstructs an image from the signals. This image represents the differences in microwave absorption inside the target. In recent years, efforts have been made to develop MTAI for the detection of breast, kidney, and prostate cancers and for brain imaging 11-15. A typical MTAI system consists of a short-pulsed microwave source, a single transducer scanning subsystem, and an acoustic signal detection subsystem. Usually, the pulse width of a single microwave pulse is at hundreds of nanoseconds (ns), but recently less than 100 ns microwave pulses were tested 16-19. The microwave source frequencies used for thermoacoustic imaging are 100, 108, and 434 MHz, and 1.2, 2.45, 6, and 9.4 GHz. Peak power of microwave source ranges from 5 kW to 350 kW 20-23.

MTAI is a potential alternative for early pancreatic cancer detection because microwave radiation has higher tissue penetration depth than light due to different contrast mechanisms. However, the difference of thermoacoustic response in the microwave-frequency between malignant tissues and healthy pancreatic tissues is low [24, Figure S1 of Supplement], limiting potential applications of MTAI in discriminating early-stage pancreatic tumors. One possible solution to this problem is to utilize exogenous contrast agents to increase the local dielectric loss in the tumors. For the past several decades, Fe_3_O_4_ nanoparticles with good biocompatibility and clinical availability have been studied as contrast agents in MRI. Fe_3_O_4_ nanoparticles also have high microwave absorption and may be advantageous in MTAI modalities 25.

Previous studies have shown that cancerous pancreatic tissue contains galectin-1 (Gal1) that is highly expressed in the cell membrane and stroma but rarely found in normal pancreatic tissues 26-38. Gal1 overexpression in tumors is a marker of malignant tumor progression and is often related to cancer metastasis. Thus, Gal1 might have utility as a target for early detection of pancreatic tumors 28- 31.

**Figure 1:**
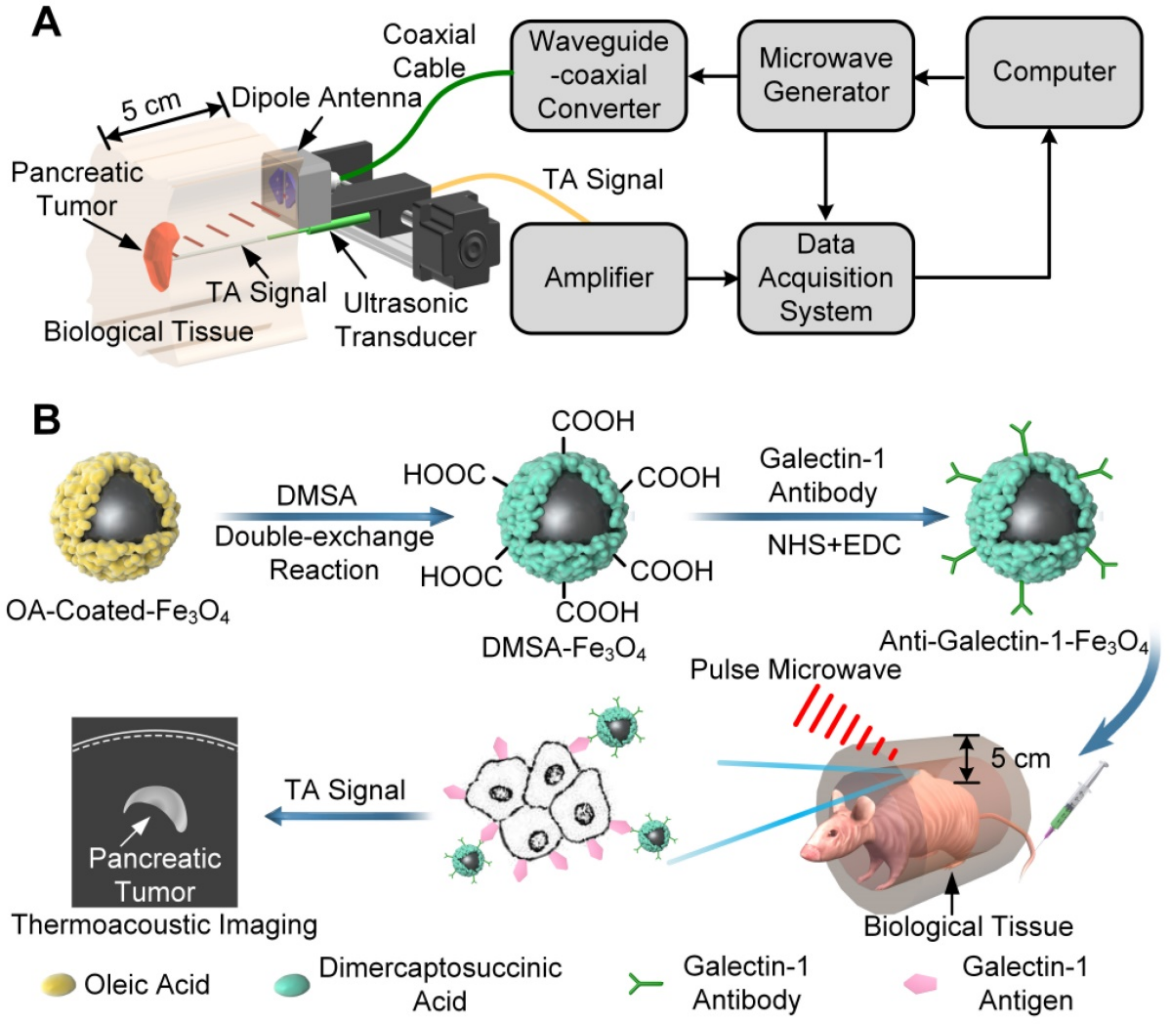
** A,** Schematic representation of MTAI. **B,** Schematic illustration of the enhanced MTAI of pancreatic tumors in a nude mouse model with anti-Gal1-Fe_3_O_4_ nanoparticles. DMSA-Fe_3_O_4_ was obtained via a double-exchange reaction of OA-coated-Fe_3_O_4_ with iron DMSA. Gal1 antibody was then coated on the surface of DMSA-Fe_3_O_4_ via an amide condensation reaction for cancer cell targeting. Anti-Gal1-Fe_3_O_4_ nanoparticles were recognized by Gal1 antigens on tumor cell membranes and accumulated in the pancreatic tumor. Tumor regions exhibit stronger microwave absorption and higher contrast relative to surrounding tissues because of the excellent electromagnetic absorption performance of anti-Gal1-Fe_3_O_4_ in MTAI.

Currently, it is challenging to use MTAI systems in the clinical settings because of the use of a bulky antenna 36, 37, that allows very limited access to the tissue/organ sites for MTAI imaging. In this study, we developed a system with a portable antenna in which MTAI could be realized in the reflection-mode, allowing its use for imaging many tissues/organ sites (Figure 1A). Also, the size of the portable microwave generator was only 42 × 30 × 18 cm^3^, and could be conveniently utilized in an ambulance or bedside. The feasibility of MTAI early pancreatic cancer detection was investigated in a nude mouse heterozygosity model with pancreatic ductal adenocarcinoma (BxPC-3). We utilized Gal1 as a molecular target and DMSA-Fe_3_O_4_ nanoparticles as a probe for early stage pancreatic tumor detection with an MTAI schematically shown in Figure 1A. DMSA-Fe_3_O_4_ nanoparticles were labeled with a Gal1 antibody to construct anti-Gal1-Fe_3_O_4_ (Figure 1B). A vector network analyzer was used to measure the microwave absorption performance of anti-Gal1-Fe_3_O_4_ nanoparticles. The targeting ability of anti-Gal1-Fe_3_O_4_ nanoparticles for pancreatic tumors was verified with fluorescence imaging. anti-Gal1-Fe_3_O_4_ nanoparticles could bind to pancreatic tumors and increase the local dielectric properties, boosting the microwave absorption in the tumors and, in turn, the MTAI signal. For *in vitro* experiments, we used excised pancreatic tumors (subcutaneous model, 1-7 mm in diameter) with inclusions containing agar at increasing depths, and compared the effect of anti-Gal1-Fe_3_O_4_ and DMSA-Fe_3_O_4_ nanoparticles on the MTAI signal. Tiny pancreatic tumors in deep tissue were identified by MTAI with high fidelity. Next, a murine model of pancreatic cancer (~3 mm in diameter tumors) was imaged with MTAI before and after intravenous injection of anti-Gal1-Fe_3_O_4_ nanoparticles. The results showed that MTAI could identify pancreatic tumors of 5 mm compared to the minimum diameter of ~10 mm achieved by other clinical nondestructive imaging methods (e.g., USI, CT, and MRI). Thus, MTAI has great potential for the detection of early stage pancreatic cancer.

## Materials and Methods

### Synthesis and characterization of anti-Gal1-Fe_3_O_4_ nanoparticles

The synthesis of anti-Gal1-Fe_3_O_4_ nanoparticles for pancreatic cancer imaging was performed following the procedure described in an earlier report 32. Oleic acid (OA)-Coated Fe_3_O_4_ (20 mg, Shanghai So-Fe Biomedical Co., Ltd.) was dissolved in 2 mL methylbenzene, and di-mercapto-succinic acid (DMSA) (20 mg) was dissolved in 2 mL dimethyl sulfoxide (DMSO). These solutions were then mixed at room temperature under magnetic stirring for 24 h, then Ethyl acetate was added and stirred further for concentration and precipitation. For the formation of the DMSA-Fe_3_O_4_, it was collected by an electromagnet and adjusted to 1 mg/mL. 2.5 µL N-hydroxy succinimide (NHS) (10 mg/mL); 5 µL 1-(3-dimethylaminopropyl)-3-ethylcarbodiimide hydrochloride (EDC) (10 mg/mL) were then dissolved in the solution of DMSA-Fe_3_O_4_ (DMSA-Fe_3_O_4_ nanoparticles, 1 mL), and sodium bicarbonate (NaHCO_3_) was added to adjust the pH value to an appropriate range (8.0~8.4) suitable for amide reaction. Next, the mixture was stirred for 30 min at room temperature (25 °C) and 20 µL Gal1 polyclonal antibody (ab154351, Immunogen: Recombinant fragment corresponding to a region within amino acids 1-135 of human Gal1, Abcam, Cambridge, MA, USA) was added. The mixture was kept at 4 °C for 12 h. The final product was collected by centrifugation and stored at 4 °C. anti-Gal1-Fe_3_O_4_ nanoparticles were characterized for particle size, zeta potential, ultraviolet spectrum, infrared spectrum, complex permittivity, and TA signal intensity. The sample (0.0001 mg/mL) was evenly distributed on the double-layer copper net, and was placed in the incubator for 24 h at 50 °C. The size of the nanoparticles was measured using a high-resolution transmission electron microscope (JEM-1400PLUS, HRTEM Company, Japan). The nanoparticles were diluted to ~0.1 mg/mL and slowly injected it into a clean quartz cuvette, and characterized by dynamic light scattering (DLS, Malvern Zetasizer Nano-ZS 90, UK). The optical characteristics of the nanoparticles were investigated by UV-vis absorption spectra (Lambda-35 UV-vis spectrophotometer, PerkinElmer, MA, USA). Fourier transform infrared (FT-IR) spectra were recorded with KBr pellets on a spectrometer (Bio-Rad FTS 6000, Bio-Rad Company, United States) at room temperature. The vector network analyzer (VNA) (AV36728-S) in our laboratory was from the 41st Institute of China Electronics Technology Group Corporation (CETC). The microwave frequency range was from 10 MHz to 26.5 GHz, and the frequency resolution was 1 Hz. A short open load transmission calibration method was used for the calibration of VNA. The purpose of the calibration was to use the difference between the predicted and actual values of three well-known standards (air, a shortcircuit and deionized water) to remove the repeatable systematic errors from the measurement. Before making a measurement, we performed a system calibration. During the experiments, it was important to confirm that the cable was stabilized and not flexed between the calibration and measurement. The air bubbles on the tip of the probe were carefully removed to ensure the accuracy. The test sample had to be thick enough to appear infinite to the probe. After calibration, the coaxial probe (3.5 mm) was slowly inserted into the 1 cm depth of 1mg/mL sample and the complex relative permittivity at different frequencies was measured. The DMSA-Fe_3_O_4_ powder was made into a round sheet of 10 mm diameter by a tablet press and put into a coaxial device to measure the permeability. The TA signal intensity of anti-Gal1-Fe_3_O_4_ nanoparticles was measured qualitatively by a self-made water immersion probe connected to an oscilloscope (Tektronix DPO5204, Beaverton, United States).

### MTAI system

In this study, the repetition frequency of the home-made microwave generator (Pulse width: 550 ns; Peak power: 70 kW) was 20 Hz and the size was 42 × 30 × 18 cm^3^. The microwave generator consisted of a power supply and magnetron (10 kV rated voltage, 70 kW peak power). The power supply provided the magnetron with a high voltage of about 10 kV. The magnetron generated pulse microwave under the power supply excitation then transmitted it to the dipole antenna through the waveguide coaxial conversion system. The size of the dipole antenna output for microwave radiation was 5 × 5 cm^2^, with an energy density in a single pulse per unit area of approximately (70 kW × 550 ns)/(5 × 5 cm^2^) = 1.54 mJ/cm^2^.

An unfocused cylindrical needle ultrasonic transducer (Active-element diameter: 0.8 mm; Sensitivity: 10 nV/Pa; model: NCS-1; Manufacturing company: Institute of Acoustics, Chinese Academy of Sciences, China) with a central frequency of 1 MHz (80% bandwidth, -6 dB) was used to detect thermosacoustic signals. The acoustic pulse captured by the transducer was first amplified through a self-made voltage amplifier (Frequency Range: 30 kHz - 10 MHz; Noise Figure: 2.2 dB) with a gain of 27.8 dB. Then it was averaged 20 times by a data acquisition card (NDAQ-50614, Topelec, China) at a sampling rate of 50-M samples=s, and finally transferred to a personal computer for subsequent image processing. The limited-field-filtered back-projection algorithm was used for reconstructing the microwave absorber distribution. The projection data obtained from the ultrasonic detector was subjected to a one-dimensional Fourier transform and a convolution operation to obtain convolution filtered data in all directions, then back-projected in all directions and processed appropriately to obtain a tomographic image of the scanned object 36-38.

### Cell uptake and biodistribution of the nanoparticles

Tumor cells were seeded on a glass-bottom petri dish at a density of 10^4^ cells/plate in 0.5 mL of culture medium for 12 h. Subsequently, cy5.5/DMSA-Fe_3_O_4_ and cy5.5/anti-Gal1-Fe_3_O_4_ nanoparticles (Fe concentration of 20 μM) were added to the culture medium. After incubation for 4 h, the cells were washed three times with phosphate-buffered saline (PBS), stained with MitoTracker Green for 30 min, washed three times with PBS, and analyzed by a Zeiss LSM 510 META laser confocal scanning microscope imaging system (Germany).

To examine whether anti-Gal1-Fe_3_O_4_ nanoparticle could enhance MTAI contrast of BxPC-3 cells, the cells were incubated with anti-Gal1-Fe_3_O_4_, DMSA-Fe_3_O_4_, and then washed three times with PBS. After centrifugation, the cells were injected into a capillary with a diameter of about 1 mm for MTAI.

The BxPC-3-bearing nude mice were randomly divided into two groups. Nude mice in groups 1 and 2 were intravenously injected with cy5.5/DMSA-Fe_3_O_4_ and cy5.5/anti-Gal1-Fe_3_O_4_ nanoparticles (Fe drug 2.5 mg/kg). Fluorescence imaging was performed at different time points (1, 3, 6, 12, and 24h post-injection) with an infrared imaging system (Odyssey LI-COR, USA).

### Imaging of the *in vivo* heterozygosity model

Next, we sought to determine whether anti-Gal1-Fe_3_O_4_ could be used for tiny pancreatic tumor detection *in vivo*. In clinical practice, the preferred examination for detecting the pancreas is in a supine position. The abdominal ultrasound probe was placed at the xiphoid process for detection. The ultrasound signal penetrated abdominal muscles and then from the liver to the pancreas. A heterozygosity model was built to investigate clinical translation at deeper imaging depths in humans by adding biologic tissue with different thicknesses (chicken breast and liver) atop the pancreatic tumor skin of nude mice. The anti-Gal1-Fe_3_O_4_ nanoparticles were employed in MTAI for tiny pancreatic tumor detection with a dosage of 2.5 mg [Fe]/kg body weight.

The anesthetized nude mice were fixed horizontally in a tank filled with mineral oil. To prevent the asphyxia of nude mice, the head and forelimbs were always isolated from the oil by rubber tubes, and oxygen and isoflurane were continuously supplied. The non-focusing cylindrical ultrasonic transducer and dipole antenna fixed on the stepping motor system were immersed in mineral oil, and one-dimensional linear scanning was carried out at a distance of 1 cm in the skin of heterozygosity model to capture TA signal.

### Animal models

The BXPC-3 cells in the logarithmic growth stage were harvested, centrifuged, and re-suspended in PBS. Subsequently, the cells were washed twice with PBS to remove serum. The cell concentration was adjusted to 1 × 10^6^ / mLwith PBS and 0.1 mL of cells were injected subcutaneously into the back of nude mice. The growth of pancreatic tumors was monitored and the diameter was recorded every day. Nude mice with a tumor diameter of 5mm were used in the subsequent experiments. All animal experiments were performed following a protocol approved by the Institutional Animal Care and Use Committee of South China Normal University. Four pancreatic tumor nude mice (BALB/c; provided by the Animal Experimental Center of Southern Medical University; weight range, 25.5-26.8 g) were initially anesthetized by using a mixture of bromethol (Nanjing AIBI Bio-technology Co., LTD, China) and tert-Amyl alcohol (Shenzhen Huarong Chemical Industry Trade Co., Ltd., China) at a dose of 240 mg/kg. TA and US images were collected before and after a 0.1-mL intravenous injection of DMSA-Fe_3_O_4_ and anti-Gal1-Fe_3_O_4_ nanoparticles with a dosage of 2.5 mg [Fe]/kg body weight. Throughout the experiment, full anesthesia was maintained with isoflurane gas, administered at 1 L/min of oxygen with 0.75% of isoflurane. Simultaneously, both heart rate and saturation of peripheral oxygen were monitored by using a pulse oximeter (AM-807, Medxing Medical, China). After imaging, animals were euthanized with an overdose of pentobarbital (Sleepaway; Huaxia Animal Health).

### Statistical analysis

The Statistical analysis and graphic display of data were performed by using Origin software (version 8.50 for Windows; Origin Lab Corporation, Northampton, United States). All values were reported as mean 6 standard error. P values were computed from a one-tailed distribution and were considered to indicate a significant difference if <0.05. Thermoacoustic signal enhancement was defined as the ratio between post- and pre-injection signal amplitudes from pancreatic tumor regions. Thermoacoustic contrast was defined as the ratio between signal amplitudes from pancreatic tumors and tissue background. ImageJ (1.5.2q, National Institutes of Health, USA) was used to compute the SNR of TA images. Oval tool is used to calculate the average gray value of the tumor area and the blank background. The linear SNR was calculated by the ratio of the average gray value of the tumor area to the average gray value of the blank background. All statistical analyses were performed by using software (Matlab 2016; MathWorks) with statistical tools (Statistics Toolbox; MathWorks).

## Results

**Figure 2:**
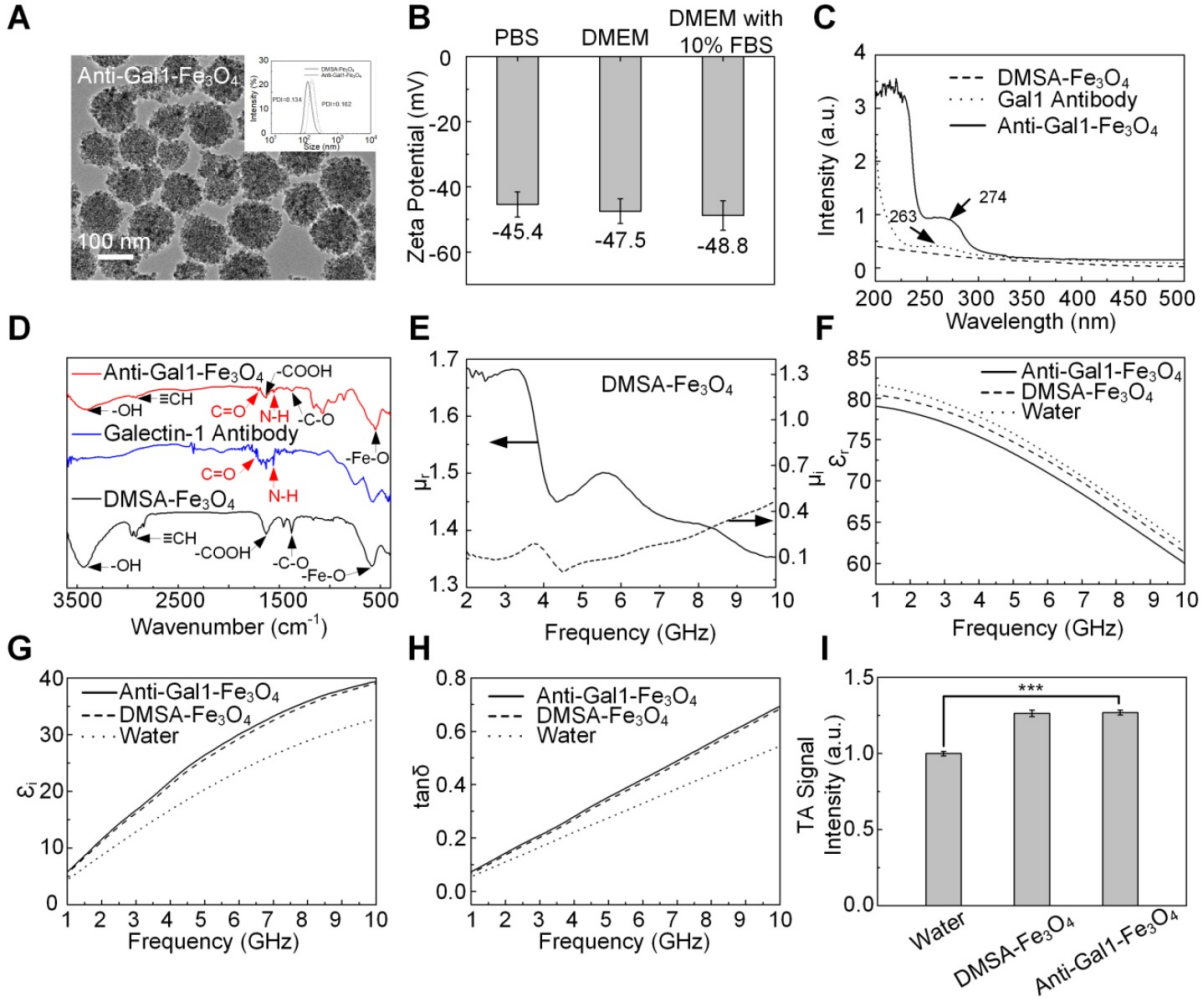
** Characterization of anti-Gal1-Fe_3_O_4_ nanoparticles. A,** Morphology of anti-Gal1-Fe_3_O_4_ nanoparticles was captured by TEM. The hydrodynamic diameter distribution was recorded for DMSA-Fe_3_O_4_ nanoparticles and anti-Gal1-Fe_3_O_4_ nanoparticles by DLS. **B,** Zeta potential for DMSA-Fe_3_O_4_ nanoparticles and anti-Gal1-Fe_3_O_4_ nanoparticles. **C,** UV Absorption spectra of DMSA-Fe_3_O_4_ nanoparticles (dashed line) and anti-Gal1-Fe_3_O_4_ nanoparticles (solid line). **D,** Fourier transform infrared spectroscopy of DMSA-Fe_3_O_4_ nanoparticles (black line), Galectin-1 antibody (blue line) and anti-Gal1-Fe_3_O_4_ nanoparticles (red line). **E,** Real (solid line) and imaginary (dashed line) parts of the permeability of DMSA-Fe_3_O_4_ nanoparticles. **F, G, H**, Real parts, imaginary parts, and tangent value of the complex relative permittivity of water, DMSA-Fe_3_O_4_ nanoparticles, and anti-Gal1-Fe_3_O_4_ nanoparticles aqueous suspension with mass concentrations of 1 mg/mL. **I,** MTAI signal intensity of DMSA-Fe_3_O_4_ and anti-Gal1-Fe_3_O_4_ nanoparticles with 1 mg/mL. The error bars represent triplicate samples and measurements (***P<0.001).

Galectin-1-targeted TA nanoprobe was composed of Gal1 antibody for targeting pancreatic tumors and a microwave absorber of Fe_3_O_4_ nanoparticles for enhancing TA signal. The physical size of the product was characterized by transmission electron microscopy (TEM) and dynamic light scattering (DLS). The anti-Gal1-Fe_3_O_4_ nanoparticles exhibited uniform and spherical nanostructures with a diameter of around 120 nm. The hydrodynamic diameter was determined by DLS, indicating the average value of 142 nm with a polydispersity index (PDI) of 0.162 (Figure 2A). Anti-Gal1-Fe_3_O_4_ nanoparticles are negatively charged and existed in monodispersed form. The nanoparticles with the same charge repelled each other and were difficult to aggregate, ensuring the dispersion of nanoparticles in the solution. The zeta potential of anti-Gal1-Fe_3_O_4_ nanoparticles was -45.4 ± 3.25 mV in PBS, -47.5 ± 3.16 mV in Dulbecco's Modified Eagle's Medium (DMEM) and -48.8 ± 3.07 mV in DMEM with 10% fetal bovine serum (FBS), suggesting that the nanoparticles were stable in normal physiological solutions (Figure 2B) 38, 39. To verify that the Gal1 antibody was successfully conjugated with the Fe_3_O_4_ nanoparticle, a Lambda-35 UV-vis (PerkinElmer, MA, USA) spectrophotometer was used to test the optical absorption spectrum of nanoparticles. The results demonstrated that DMSA-Fe_3_O_4_ nanoparticles conjugated with the Gal1 antibody had a characteristic UV absorption peak of protein at 250-300 nm (Figure 2C). The Fourier transform infrared spectroscopy of DMSA-Fe_3_O_4_ Gal1 antibody and anti-Gal1-Fe_3_O_4_ further verified the formation of anti-Gal1-Fe_3_O_4_ (Figure 2D). There were characteristic peaks of DMSA (≡CH, -COOH, and -C-O) and Fe_3_O_4_ (-OH and Fe-O) on the surfaces of anti-Gal1-Fe_3_O_4_ and DMSA-Fe_3_O_4_ and there were amide I (C=O, 1690 cm^-1^) and amide II bands (N-H, 1560 cm^-1^) on the surface of anti-Gal1-Fe_3_O_4_, but not on the surface of DMSA-Fe_3_O_4_ 60-63. The results provided evidence that Gal1 antibody had successfully conjugated with the Fe_3_O_4_ nanoparticle.

To explore the microwave absorption properties of DMSA-Fe_3_O_4_ and anti-Gal1-Fe_3_O_4_ nanoparticles, their relative dielectric constant and magnetic permeability were investigated with a vector network analyzer (AV3672B-S, the 41^st^ Institute of CETC, China). Parameters related to microwave absorption including the permeability real part (µ_r_), imaginary part (µ_i_), and permittivity real part (ε_r_), imaginary part (ε_i_), and loss tangent (tan δ) values were obtained (Figure 2E-H). The ε_i_ and tan δ for anti-Gal1-Fe_3_O_4_ showed higher values than deionized water, indicating a strong microwave absorption capacity. Anti-Gal1-Fe_3_O_4_ nanoparticles and unmodified Fe_3_O_4_ nanoparticles exhibited similar dielectric properties, indicating identical microwave absorption properties (Figure 2I). The TA signal intensity of anti-Gal1-Fe_3_O_4_ nanoparticles with 1 mg/mL was >17% higher than that of deionized water (P < 0.001). Unmodified Fe_3_O_4_ nanoparticles displayed TA signal intensity that was similar to anti-Gal1-Fe_3_O_4_ nanoparticles (P <0.001). The stability of anti-Gal1-Fe_3_O_4_ nanoparticles was confirmed, as displayed in Figure S3 (see supplementary material for details).

**Figure 3:**
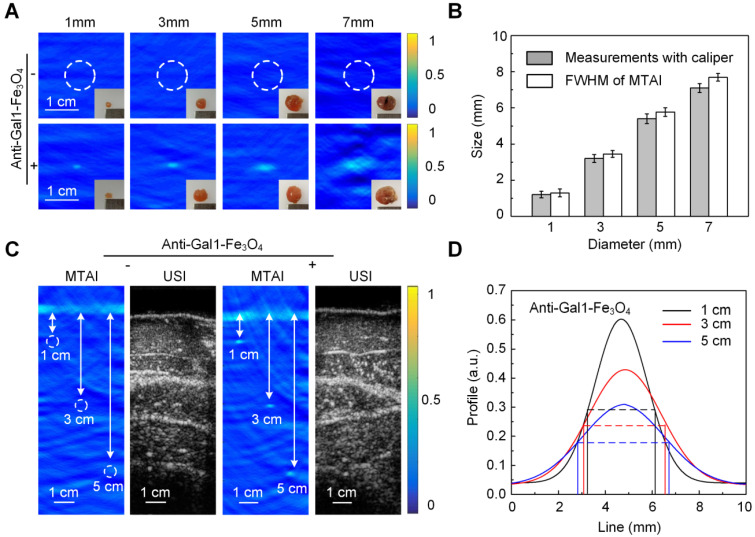
** MTAI of pancreatic tumor-mimicking phantoms with increasing tumor size and/or imaging depth. A,** MTAI of pancreatic tumors as a function of size. **B,** FWHM of MTAI taken along the dashed lines in **A**. **C,** MTAI and US images of one slice through the tumors. **D,** Three 1D profiles from the dashed lines in **C**. Tumors injected with anti-Gal1-Fe_3_O_4_ nanoparticles show significantly higher TA signals than the control specimens, suggesting that the improvements in contrast were due to enhanced microwave absorption. All MTAI and USI have the same scale bar of 1 cm.

The excised pancreatic tumors with different sizes were used as phantoms (in Figure 3). We used a subcutaneous tumor model where BXPC-3 pancreatic tumor cells were inoculated in the back of male BALB/c nude mice. Tumors with different sizes were excised from mice with and without intravenous injection of anti-Gal1-Fe_3_O_4_ nanoparticles at the dose of 2.5 mg Fe/kg of body weight. Pancreatic tumors mimicking phantoms with diameters of 1.0 mm, 3.1 mm, 5.0 mm, and 7.2 mm were imaged to explore the resolving power of MTAI and the tumors with anti-Gal1-Fe_3_O_4_ are could be seen in the TA images. The average FWHM of the four 1D profiles were 1.2 mm ± 0.14, 3.1 mm ± 0.17, 5.2 mm ± 0.22, 7.2 mm ± 0.25, which were comparable to the sizes of the dissected pancreatic tumors as measured with a caliper (Figure 3A, B). These results demonstrated that anti-Gal1-Fe_3_O_4_ could enhance MTAI contrast in pancreatic tumor mimicking phantoms and that MTAI could distinguish phantoms less than 10 mm in diameter.

Next, the feasibility of MTAI for identifying tiny pancreatic tumors in deep tissue was investigated. The pancreas is generally about 4 centimeters deep from the skin. To simulate an actual imaging scenario, pancreatic tumor mimicking phantoms with diameters of 3.0 mm were covered with a layer of hybrid biological tissue. Figure 3C shows MTAI of pancreatic tumors at different depths of 1 cm, 3 cm, and 5 cm in the transverse plane. The TA signal from pancreatic tumor-mimicking phantoms with anti-Gal1-Fe_3_O_4_ nanoparticles was more prominent than that of pancreatic tumors without anti-Gal1-Fe_3_O_4_ nanoparticles. Although the imaging depth was increased, the average FWHM of the 1D profiles were 3.1 mm ± 0.21, 3.3 mm ± 0.18, 3.4 mm ± 0.19 at a depth of 1 cm, 3 cm, and 5 cm, respectively (Figure 3D), which were comparable to the caliper-measured sizes of the pancreatic tumor mimicking phantoms. Thus we have demonstrated that MTAI has the potential for imaging tiny pancreatic tumors in deep tissues with high fidelity.

**Figure 4:**
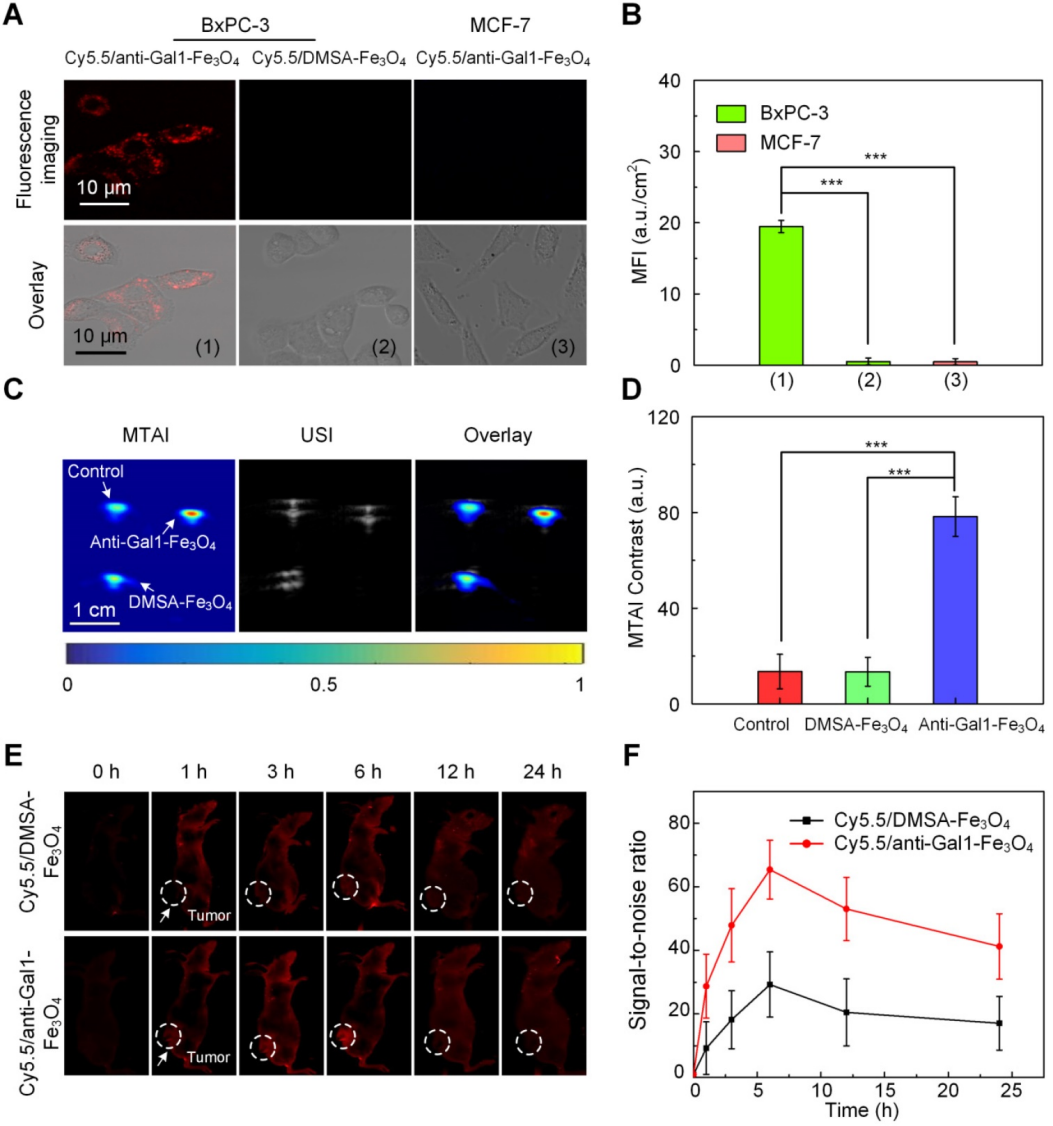
** Evaluation of the targeting ability of anti-Gal1-Fe_3_O_4_ nanoparticles. A,** CLSM images of pancreatic cancer cells (BxPC-3) and breast cancer cells (MCF-7). **B,** Mean fluorescence intensity (MFI) in the interior cell region. **C,** MTAI, USI and overlay images of BxPC-3 in capillaries. **D,** MTAI contrast of BxPC-3 in capillaries. **E,** *In vivo* fluorescence imaging of a BxPC-3 murine model upon injection with Cy5.5/DMSA-Fe_3_O_4_ nanoparticles and Cy5.5/anti-Gal1-Fe_3_O_4_ nanoparticles at different time intervals. **F,** Fluorescence signal-to-noise ratio (SNR) in tumors upon injection with Cy5.5/DMSA-Fe_3_O_4_ nanoparticles and Cy5.5/anti-Gal1-Fe_3_O_4_ nanoparticles at different times (**P<0.01; ***P<0.001).

Gal1 is highly expressed in pancreatic tumor cells and acts as a receptor for extracellular matrix proteins upon exposure to the Gal1 antibody 26-31. To test whether anti-Gal1-Fe_3_O_4_ nanoparticles (Cy5.5-labelled) could specifically target pancreatic tumor cells (BxPC-3 cells), we incubated the nanoparticles with BxPC-3 cells overexpressing Gal1 on the surface; MCF-7 cells with low expression of Gal1 was used as a control. As an additional control, we also incubated BxPC-3 cells with Cy5.5-labelled DMSA-Fe_3_O_4_ nanoparticles (without the Gal1 antibody) and subjected all groups to the same treatment protocol. Using confocal microscopy, we detected fluorescence signal on the membranes of BxPC-3 cells incubated with anti-Gal1-Fe_3_O_4_ nanoparticles, while there was very minimal signal in the MCF-7 and BxPC-3 cells incubated with DMSA-Fe_3_O_4_ nanoparticles (Figure 4A, B). These results indicated that the anti-Gal1-Fe_3_O_4_ nanoparticles were specific to BxPC-3 cells and that targeting was mainly mediated by the interaction between Gal1 and Gal1 antibody.

To examine whether anti-Gal1-Fe_3_O_4_ nanoparticles could enhance MTAI contrast, BxPC-3 cells incubated with anti-Gal1-Fe_3_O_4_ nanoparticles and with saline were imaged. BxPC-3 cells incubated with DMSA-Fe_3_O_4_ nanoparticles served as an additional control. BxPC-3 cells incubated with anti-Gal1-Fe_3_O_4_ nanoparticles showed strong TA signal, while minimal TA signal was observed upon incubation of cells with saline or DMSA-Fe_3_O_4_ nanoparticles. The results indicated that anti-Gal1-Fe_3_O_4_ nanoparticles could specifically enhance MTAI contrast of BxPC-3 cells (Figure 4C, D).

We investigated the preferential accumulation of Cy5.5/anti-Gal1-Fe_3_O_4_ nanoparticles in tumor regions at various time points post-injection with an *in vivo* fluorescence imaging system. The tumors of Cy5.5/anti-Gal1-Fe_3_O_4_ nanoparticle-treated nude mice had much higher fluorescence intensities indicating better accumulation compared to mice treated with Cy5.5/DMSA-Fe_3_O_4_ nanoparticles at all time points (Figure 4E). The fluorescence intensity reached a maximum at 6 h post-injection for both nanoparticles. At this time, the signal-to-noise ratio (SNR) for Cy5.5/anti-Gal1-Fe_3_O_4_ nanoparticles-treated nude mice tumors was 2.1-fold higher compared with Cy5.5/DMSA-Fe_3_O_4_ nanoparticles treatment (Figure 4F). Overall, these observations demonstrated the specific targeting properties of the anti-Gal1-Fe_3_O_4_ nanoparticles toward pancreatic tumors.

**Figure 5:**
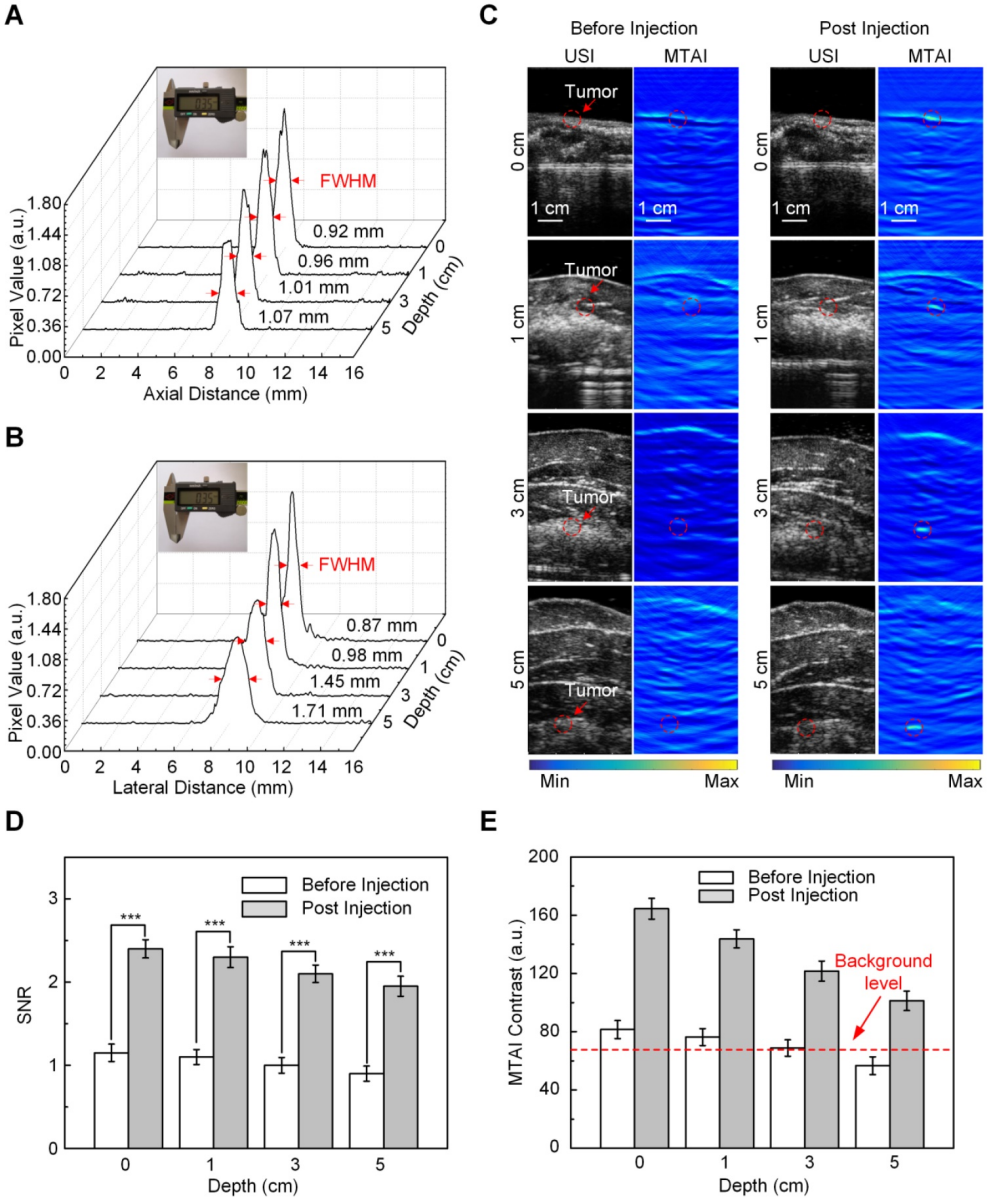
***In vivo* MTAI of pancreatic tumors in a heterozygosity model. A,** Axial resolution of MTAI. **B,** Lateral resolution of MTAI.** C,** MTAI of pancreatic tumors covered with a layer of chicken breast and liver of different thicknesses on top of the mouse skin surface at increasing depths* in vivo*. **D,** Signal-to-noise ratio (SNR) in pancreatic tumors before and after injection with anti-Gal1-Fe_3_O_4_ at increasing depths. **E,** MTAI contrast of the pancreatic tumors. The error bars are based on triplicate samples. All MTAI and USI have the same scale bar of 1 cm (**P<0.01, ***P<0.001).

Next, we tested the ability of MTAI with anti-Gal1-Fe_3_O_4_ nanoparticles to identify tiny pancreatic tumors less than 5 mm in diameter within deep tissues. To simulate actual imaging, *in vivo* heterozygosity models were built by covering pancreatic tumors in nude mice with biologic tissues. Resolution experiments were performed to verify the imaging capability of the MTAI system in the simulated imaging scenarios. A 0.35 mm diameter copper wire was used as the microwave absorber to test the axial and lateral resolution of the MTAI system at different tissue depths. The axial resolution, which is determined by time-resolved ultrasonic detection, could be estimated with the following formula: *c*/2*f*, where *c* is the speed of TA signal in oil (1500 m/s), and *f* is the main frequency of ultrasonic transducer (1 MHz). The theoretical axial resolution for the transducer used in our experiments was 0.75 mm. The actual axial resolution of MTAI at depths of 0 cm, 1 cm, 3 cm, and 5 cm was 0.92 ± 0.07 mm, 0.96 ± 0.09 mm, 1.01 ± 0.12 mm, 1.07 ± 0.11 mm (P = 0.001) (Figure 5A), respectively. The lateral resolution was decreased along with the increase of imaging depth. The axial resolution of MTAI at four depths of 0 cm, 1 cm, 3 cm and 5 cm was measured to be 0.87 ± 0.06 mm, 0.98 ± 0.10 mm, 1.45 ± 0.13 mm, 1.71 ± 0.15 mm (standard error of the mean) (P = 0.001) (Figure 5B). Pancreatic tumors at three different depths [(thin human abdominal wall (0.5 cm-thick chicken breast + 0.5 cm-thick liver), normal human abdominal wall (1.5 cm-thick chicken breast + 1.5 cm-thick liver), and thick human abdominal wall (2.5 cm-thick chicken breast + 2.5 cm-thick liver)] were examined with MTAI before and after anti-Gal1-Fe_3_O_4_ nanoparticle injection through the tail vein with a dosage of 2.5 mg [Fe]/kg body weight. Pancreatic tumors at all four depths were clearly visible with SNR of 2.3 ± 0.15, 2.1 ± 0.09, 1.8 ± 0.14, and 1.9 ± 0.12 (P = 0.001) at 6 h after injection (Figure 5C, D). Notably, The MTAI contrast of pancreatic tumors after injection of anti-Gal1-Fe_3_O_4_ nanoparticles increased by about 2.2- fold compared to pre-injection (standard deviation, 0.13) (Figure 5E). Generally, the dynamic range of image display was about 40-50 dB; however, the dynamic range of MTAI was less than 40 dB. During image post-processing, the TA signal was nonlinearly amplified to 50 dB to maximize differences in the display so the users could directly observe the image difference before and after the injection of nanoparticles. These results further demonstrated that the anti-Gal1-Fe_3_O_4_ nanoparticles could target the tumor site, and ultimately specifically enhanced the MTAI tumor contrast. Thus, MTAI could be a potential alternative for the detection of tiny pancreatic tumors.

**Figure 6:**
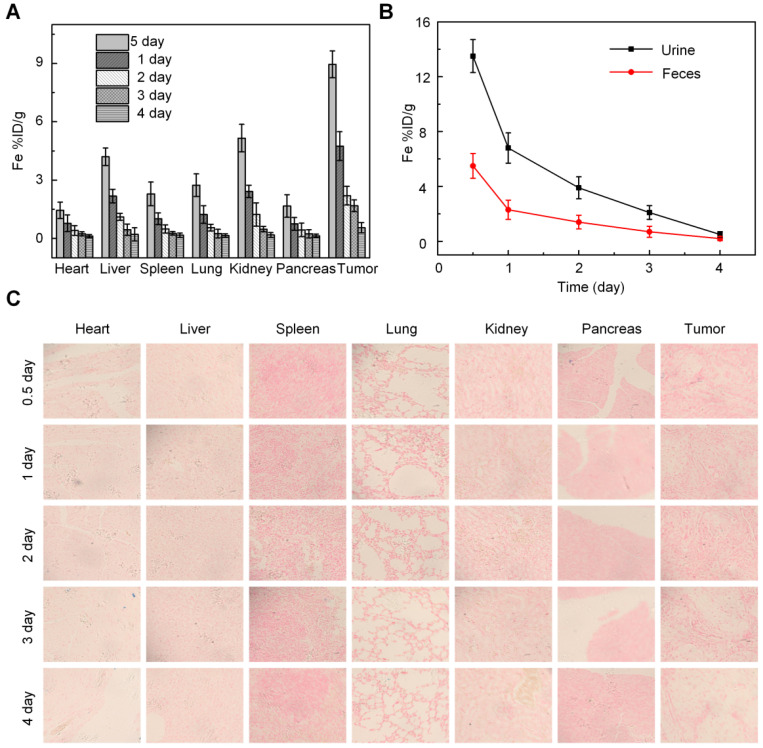
***In vivo* studies. A,** Determination of Fe content in different tissues by ICP-MS.** B,** Excretion profile of anti-Gal1-Fe_3_O_4_. **C,** Prussian blue staining of various major organs after administration of anti-Gal1-Fe_3_O_4_ (0.5 mg/mL). The representative specimens were at ×400 magnification.

We investigated the *in vivo* behavior of anti-Gal1-Fe_3_O_4_ by measuring Fe levels in the tumors, various organs, urine and feces samples using inductively coupled plasma mass spectrometry (ICP-MS). Biodistribution data of anti-Gal1-Fe_3_O_4_ at different time intervals post-injection revealed relatively low accumulation of anti-Gal1-Fe_3_O_4_ in the heart and lung (Figure 6A). The accumulation of anti-Gal1-Fe_3_O_4_ in the liver and spleen was determined to be ~1.8 and ~1.2% ID at 24 h post-injection and further decreased to low levels after 3 days. High levels of the Ferrum element were observed in the tumor with about 4.8% ID at 24 h post-injection. This was likely due to the DMSA coating on the surface of Fe_3_O_4_, which could delay their macrophage clearance and escape from the reticuloendothelial systems (RES) during blood circulation and favor accumulation in the tumor by the dual targeting effect of the EPR and subsequent targeted binding with the Galectin-1 antigen on tumors. The feces and urine were also collected to study the possible clearance pathway of anti-Gal1-Fe_3_O_4_ (Figure 6B). High levels of Fe were detected in both urine and feces, indicating that anti-Gal1-Fe_3_O_4_ could be excreted through both renal and fecal routes, and very little retention of the remaining Fe-species was observed in the mouse body after 4 days. This observation was consistent with low levels of anti-Gal1-Fe_3_O_4_ in the liver, spleen, and kidney.

*In vivo* targeting efficiency of anti-Gal1-Fe_3_O_4_ after intravenous injection was evaluated by Prussian blue staining at different times. Within 12 h post-injection, due to the effective tumor accumulation and relatively less excretion, anti-Gal1-Fe_3_O_4_ was mainly distributed in the pancreatic tumor, and was rarely present in other organs such as heart, liver, spleen, lung, kidney and pancreas (Figure 6C), which was consistent with the results of ICP-MS.

## Discussion

The gold standard for early detection of pancreatic cancer, Endoscopic Ultrasound (EUS), has a sensitivity of > 90% and is superior to the non-invasive MRI, USI and CT 40, 41. However, EUS is an invasive imaging procedure and complications such as aspiration, bleeding, perforation of the digestive tract, and cardiovascular accidents may occur during the process 42-44. In this context, MTAI of biological tissue has attracted considerable interest because it combines the spatial resolution of ultrasound and the contrast of microwave absorption 45, 46. MTAI is non-invasive and has a lower risk compared with EUS. We demonstrated the feasibility of non-invasive *in vivo* detection of small pancreatic tumors in a heterozygous model using MTAI following anti-Gal1-Fe_3_O_4_ nanoparticle injection. Fe_3_O_4_ nanoparticles have been used extensively in humans with low toxicity 47, 58 and can be safely cleared from the body via the skin, bile, and liver 49-58. Cell death was analyzed by MTT and FACS, showing that anti-Gal1-Fe_3_O_4_ had no significant effect on pancreatic tumor cells (BxPC-3) or macrophages (RAW 264.7) after 24 h even at concentrations as high as 0.5 mg/mL (Figure S4 of Supplement). The *in vivo* effect of anti-Gal1-Fe_3_O_4_ at this dose showed little long-term toxicity in nude mice (Figure 6). An efficient generation of TA signals requires thermal confinement. In our imaging system, the microwave pulse width was 550 ns with a repetition rate of 20 Hz, which met the thermal confinement condition. Heat diffusion was negligible and temperature increment was minimal. The microwave fluence on the skin was less than 10 mJ/cm^2^, and could be increased by a factor of 12 while still conforming to the maximal permissible exposure allowed by the American National Standards Institute limits (20 mJ/cm^2^) 59. TA signal strength and imaging depth separately depend on microwave absorption and fluence, and thus a higher concentration of contrast agents would likely enable better pancreatic cancer detection with even higher contrast values. Stronger microwave fluence would also enable tiny pancreatic cancer detection with even deeper imaging depths.

Although we conducted preliminary studies of the uptake dynamics of anti-Gal1-Fe_3_O_4_ nanoparticles in the pancreatic tumors of nude mice, further pharmacokinetic/pharmacodynamic studies are required including dose escalation studies. We performed initial feasibility studies, but better results might be achieved by tuning the nanoparticle parameters (e.g., shape, size). Additional studies are also needed to optimize microwave frequency.

The widely used microwave absorbers in TA imaging can be basically divided into two major categories according to their microwave loss mechanism. Nanomaterials such as carbon nanotubes, molybdenum disulfide and graphene nanoparticles are selected to be TA contrast agents based on their relatively large dielectric-loss, where the microwave absorption capability can be further improved by constructing charge disequilibrium sites such as defects on these nanomaterials 64, 65. The second category microwave absorbers are hysteresis-loss-type nanomaterials and their microwave absorption capability can be achieved by optimizing structure and components to improve their hysteresis losses.

It is often argued that imaging may not be as effective for diagnosis as biomarkers, which are at the center stage for early detection 66-69. However, imaging is always required for radiation therapy treatment planning. Given the thrust towards hypofractionation and high- dose therapy protocols, imaging becomes even more important. The imaging modality we have described in the current study may provide yet another effective tool in battling pancreatic cancer.

In summary, MTAI offers deep imaging depth and high contrast when used with anti-Gal1-Fe_3_O_4_ nanoparticles. MTAI can identify pancreatic tumors smaller than 5 mm in diameter, which is beyond the minimum size (~10 mm in diameter) achievable by other nondestructive clinical imaging methods. Thus, MTAI is an alternative for early pancreatic cancer detection.

### Practical applications

The potential clinical implications of our study are important, as the ability to visualize abdominal pancreatic tumors *in vivo* permits the translation of this technology into the clinic. Image-guided biopsy of pancreatic lesions is now the standard of care. Although the use of a standard abdominal US is helpful in a subset of patients with pancreatic cancer, it is only helpful if the patient has a morphologically abnormal pancreatic mass. To the best of our knowledge, there are no reliable methods to identify pancreatic tumors noninvasively. This combination of noninvasive detection and needle biopsy would allow clinicians to diagnose pancreatic malignancies at an early stage and direct appropriate treatment based on early detection.

## Supplementary Material

Supplementary figures and tables.

## Abbreviations

MTAI: Microwave-induced thermoacoustic imaging
USI: ultrasound imaging
CT: computed tomography
MRI: magnetic resonance imaging
Gal1: Galectin-1
OA: Oleic acid
FWHM: The average full widths at half maximum
DMSA: Dimercaptosuccinic acid
DMSO: Dimethyl sulfoxide
NHS: N-hydroxy succinimide
EDC: 1-(3-Dimethylaminopropyl)-3-ethylcarbodiimide hydrochlo-ride
NaHCO_3_: Sodium Bicarbonate
