# Supplementary material for: Pancreatic Cancer detection via Galectin-1-targeted Thermoacoustic Imaging: validation in an in vivo heterozygosity model
Source: Theranostics. 2020 Jul 14;10(20):9172–85. doi: 10.7150/thno.45994 (PMC7415802; doi:10.7150/thno.45994)
Supplement: Supplementary file 1 — Supplementary figures and tables. [file thnov10p9172s1.pdf]

Supplementary Information:

Pancreatic Cancer Detection via Galectin-1-targeted Thermoacoustic Imaging: Validation in an *in vivo* Heterozygosity Model

Huan Qin[#1, 2✉], Baohua Qin[#1, 2], Chang Yuan[1, 2], Qun Chen[1, 2], and Da Xing[1, 2✉]

1. MOE Key Laboratory of Laser Life Science & Institute of Laser Life Science, College of Biophotonics, South China Normal University, Guangzhou 510631, China;
2. Guangdong Provincial Key Laboratory of Laser Life Science, College of Biophotonics, South China Normal University, Guangzhou 510631, China;

✉ Corresponding authors: Huan Qin, Ph.D., MOE Key Laboratory of Laser Life Science & Institute of Laser Life Science, South China Normal University, Guangzhou, 510631, China. E-mail: qinghuan@scnu.edu.cn. Da Xing, Ph.D., MOE Key Laboratory of Laser Life Science & Institute of Laser Life Science, South China Normal University, Guangzhou, 510631, China. Phone: +86-20-85211436-8301; Fax: +86-20-85211436; E-mail: xingda@scnu.edu.cn.

[#]These authors contributed equally to this work.

© Ivyspring International Publisher. This is an open access article distributed under the terms of the Creative Commons Attribution (CC BY-NC) license (https://creativecommons.org/licenses/by-nc/4.0/). See http://ivyspring.com/terms for full terms and conditions.

Received: 2020.03.xx; Accepted: 2020.xx.xx; Published: 2020.xx.xx

Herein, the microwave absorption of pancreatic tumors and normal pancreatic tissues were measured by using a home-made microwave absorption device. Samples of equal volume were homogenized, an immersion transducer was used to measure microwave absorption in a fixed position, and the absorption peak was read on the oscilloscope and was normalized. As shown in Figure S1, the difference of thermoacoustic response in the microwave-frequency between malignant tissues and healthy pancreatic tissues was low, limiting applications of MTAI in discriminating early-stage pancreatic tumors. One potential solution to this problem was to utilize exogenous contrast agents to increase the local dielectric loss in the tumors.

The DMSA-Fe_3O_4 were characterized by particle size, energy spectrum, infrared spectrum, and complex permeability. The TEM results showed that DMSA-Fe_3O_4 had uniform spherical nanostructures with around 120 nm in size (Figure S2 A). The constituent elements of DMSA-Fe_3O_4 were analyzed by an energy dispersive spectroscope (EDS), as shown in Figure S2 B, the contents of ferrum (Fe, 41.51%), carbon (C, 31.44%), oxygen (O, 26.29%), and sulphur (S, 0.75%) were different. Carbon, Sulphur, and oxygen were the constituents of DMSA, Fe_3O_4 was made up of atoms of ferrum and oxygen. As an organic small molecule, the hydrodynamic particle size of DMSA is about 0.5 nm (Figure S2 C) and it had little effect on the size of DMSA-Fe_3O_4.

Next, the infra-red spectrogram was measured to further verify the success of DMSA-Fe_3O_4 synthesis, and each

characteristic peak was marked (Figure S2 D). The infra-red spectrum of DMSA-Fe_3O_4 contained the characteristic peaks of DMSA (≡CH, -COOH, and -C-O) and of Fe_3O_4 (-OH and Fe-O). Therefore, the synthesis of DMSA-Fe_3O_4 met the experimental requirements.

For evaluating the stability of nanoparticles, the storage stability was examined for DMSA-Fe_3O_4 and anti-Gal1-Fe_3O_4 for 24 hours. No obvious precipitation at normal temperature could be seen in both solutions (Figure S3 A). The stability of two kinds of nanoparticles was further evaluated by DLS analysis under simulated physiological conditions such as blood circulation. As shown in Figure S3 B and D, the polymer dispersity index (PDI) and zeta potential values of DMSA-Fe_3O_4 and anti-Gal1-Fe_3O_4 showed minimal change. The PDI of DMSA-Fe_3O_4 and anti-Gal1-Fe_3O_4 was near 0.2 because of the good dispersion after surface modification. Upon treating with PBS, DMEM, and DMEM with 10% FBS at 37 ℃, the particle size of anti-Gal1-Fe_3O_4 was comparable with the initial state. Taken together, anti-Gal1-Fe_3O_4 had good stability.

Furthermore, the constituent elements of anti-Gal1-Fe_3O_4 were analyzed by an energy dispersive spectroscope (EDS). The contents of ferrum (Fe, 34.12%), carbon (C, 46.69%), oxygen (O, 18.98%), sulphur (S, 0.75%) and Nitrogen (N, 0.08%) are different (Figure S11). In addition to carbon, sulphur and oxygen, nitrogen is a unique component of Galectin-1 antibody. Fe_3O_4 is made up of atoms of ferrum and oxygen. This result provides an evidence that Gal1 antibody has successfully modified to the Fe_3O_4 nanoparticle.

The cell viability of BXPC-3 and RAW264.7 cells treated with anti-Gal1-Fe_3O_4 (0~500 μg/mL) for 24 h have been examined to evaluate its toxicity. The results indicated that anti-Gal1-Fe_3O_4 had no obvious toxicity in vitro (Figure S4 A). Next, we investigated the *in vivo* toxicology of anti-Gal1-Fe_3O_4 in 6-week old BALB/c mice. A cohort of 20 healthy male mice were randomly divided into two groups. The group injected with saline was as control group. The other group intravenously injected with the anti-Gal1-Fe_3O_4 nanoparticle (2.5 mg Fe/kg). We recorded the body weight and behavior of the animals on a daily basis during the course of the experiment. We then performed histological analyses 7 days postinjection. Body weight in the nanoparticle-treated groups showed no statistically significant (Figure S4 B). No unusual behaviors were observed in any of the animals. These results suggest the nanoparticle had negligible toxicity *in vivo*. Upon examining hematoxylin and eosin (H&E) staining of the major organs including the liver, spleen, kidney, heart, and lung (Figure S4 C), we did not observe any noticeable organ damage. These results suggest the nanoparticle had negligible toxicity *in vivo*.

Table 1. Detailed parameters of the magnetron.

**ABRIDGED DATA**	
**Fixed frequency pulse magnetron**	
Operating frequency	3050 ± 25 MHz
Typical peak output power	72 kW
Magnet	Integral
Output	No. 10 waveguide (72.4 × 34.04 mm internal)
Coupler	Mates with NATO S.N. 5985-99-083-0058
Cooling	Forced-air
**GENERAL DATA**	
**Electrical**	
Cathode	Indirectly heated
Heater voltage	6.3 V
Heater current 6.3v	1.25 A
Heater starting current, peak value,not to be exceeded	6.0 A MAX
Cathode pre-heating time (minimum)	3.0 min
**TYPICAL OPERATION**	
**Operating Conditions**	
Heater voltage	3.8 - 5.0 V
Anode current (peak)	11 - 15 A
Pulse duration	0.55 - 1.0 μs
Pulse repetition rate	1000 pps
Rate of rise of voltage pulse	120 - 130 kV/μs
**Typical Performance**	
Anode voltage (peak)	9.4 - 10 kV
Output power (peak)	48 - 72 kW
Output power (mean)	34 - 48 W

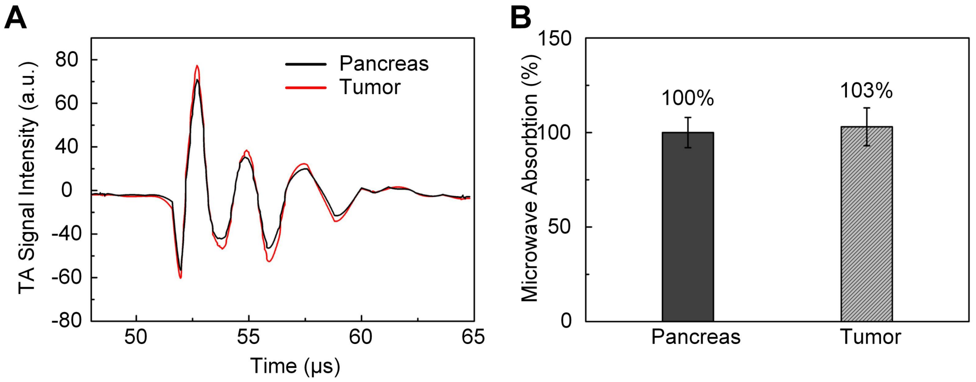

Figure S1. TA signal intensity and microwave absorption of normal pancreas and pancreatic tumors suspension.

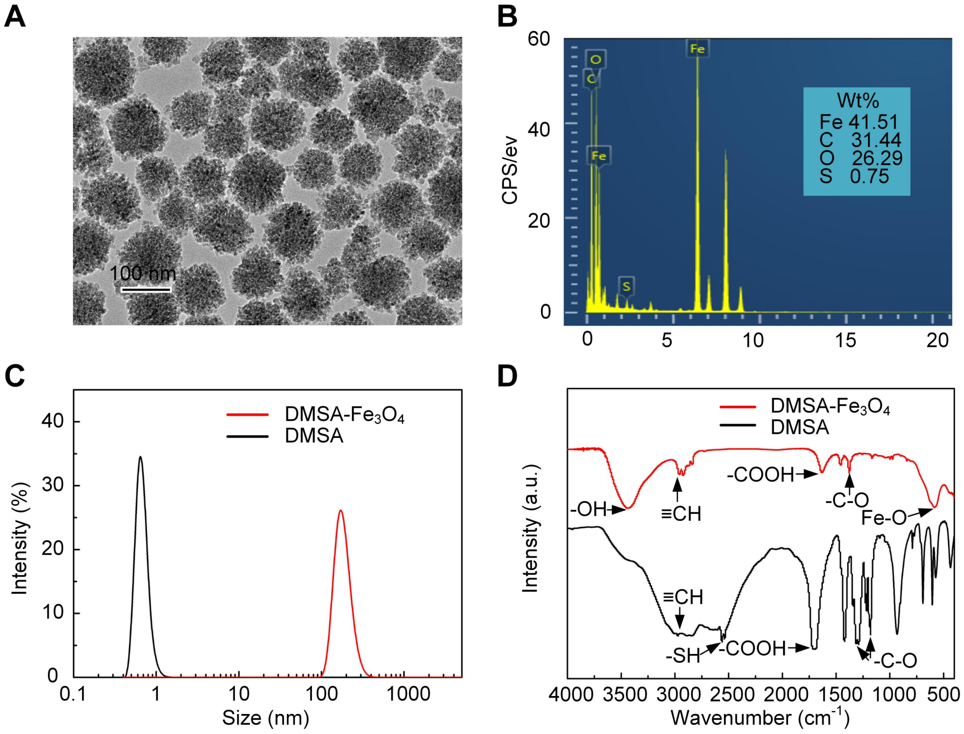

Figure S2. Nanoparticle characterization. (A) TEM images of DMSA-Fe_3O_4. (B) Energy spectrum distribution of DMSA-Fe_3O_4. (C) Size distribution of DMSA and DMSA-Fe_3O_4 determined by dynamic light scattering (DLS) at 25 °C. (D) Infra-red spectrogram of DMSA and DMSA-Fe_3O_4.

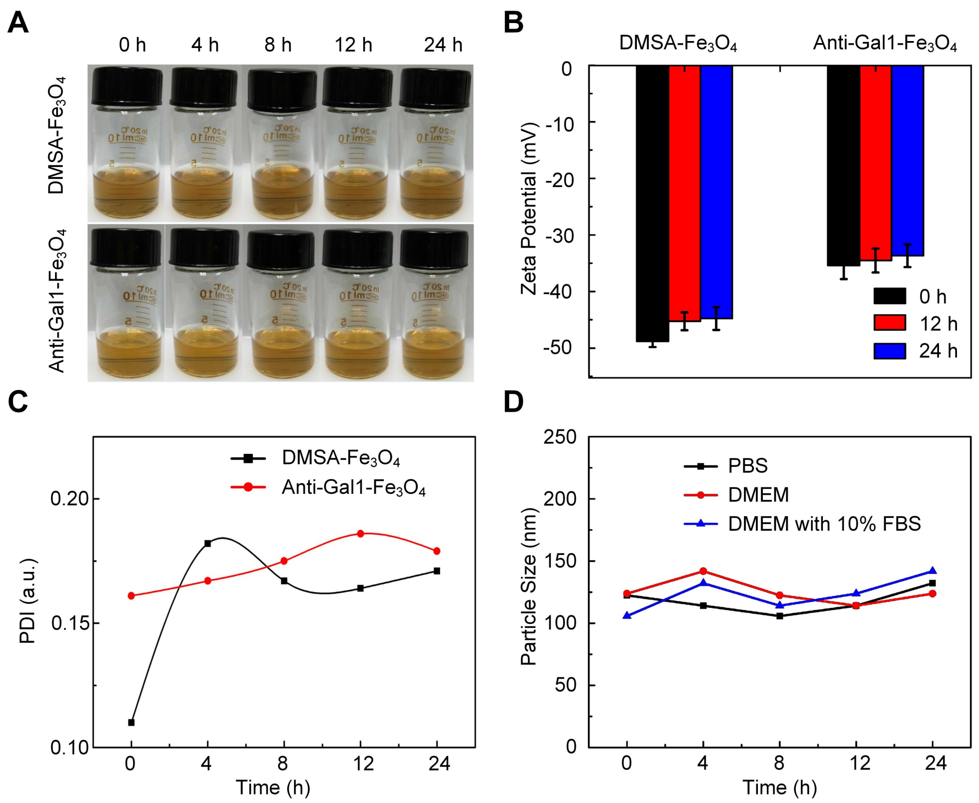

Figure S3. Stability of nanoparticles. (A) Photos of DMSA-Fe_3O_4 and anti-Gal1-Fe_3O_4 at varying times. (B) Zeta potential detection of DMSA-Fe_3O_4 and anti-Gal1-Fe_3O_4 at varying times. (C) PDI of DMSA-Fe_3O_4 and anti-Gal1-Fe_3O_4 at varying times. (D) Average hydrodynamic diameter recorded for anti-Gal1-Fe_3O_4 (500 μg/mL) upon incubation in different media at 37 °C to verify the colloidal stability of anti-Gal1-Fe_3O_4.

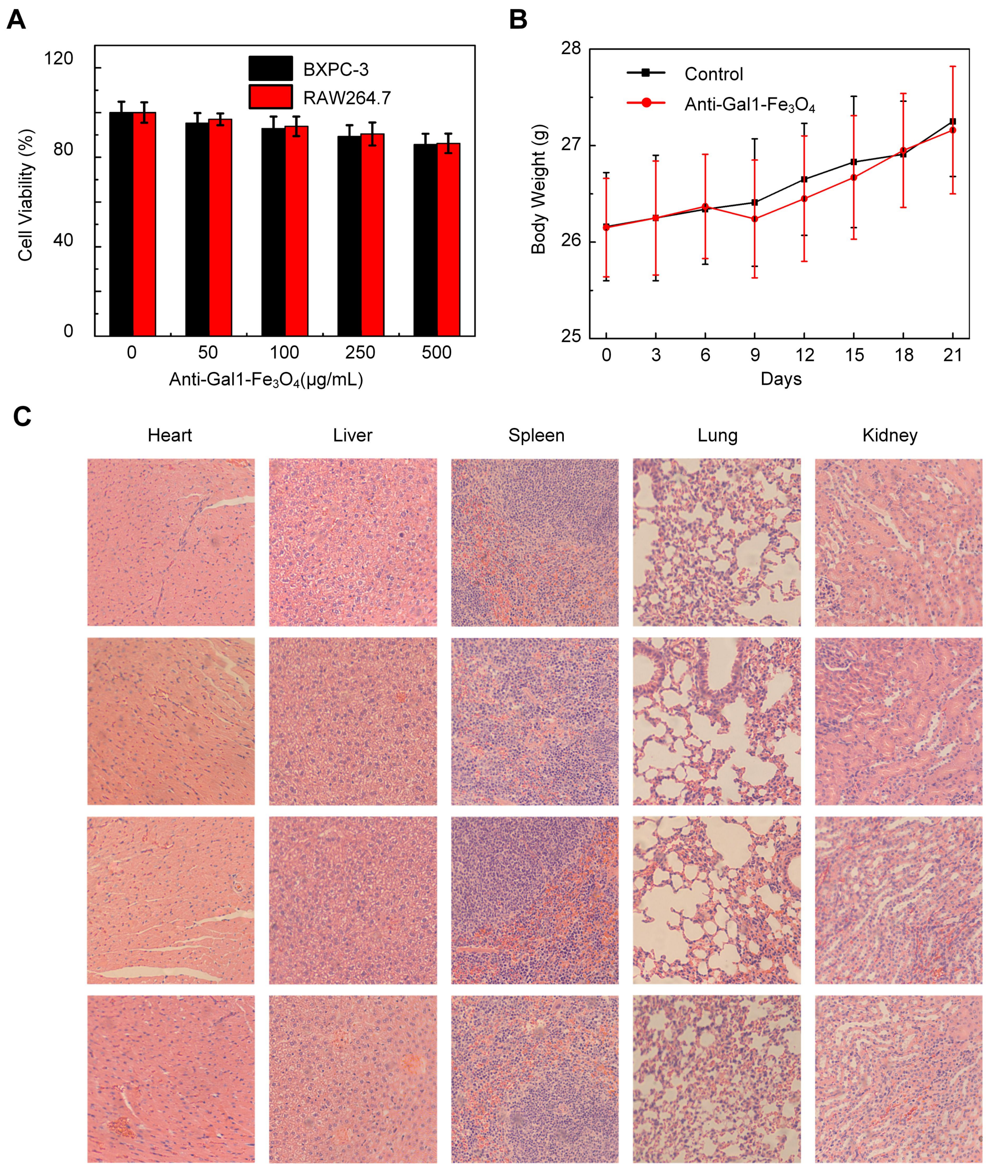

Figure S4. Biocompatibility experiment of anti-Gal1-Fe_3O_4. A, Relative cell viability of BXPC-3 and RAW264.7 cells after incubation with DMSA-Fe_3O_4 and anti-Gal1-Fe_3O_4. **B,** Bodyweight of mice after various treatments. **C,** H&E staining of the heart, liver, spleen, lung, kidney, and pancreas from different groups.

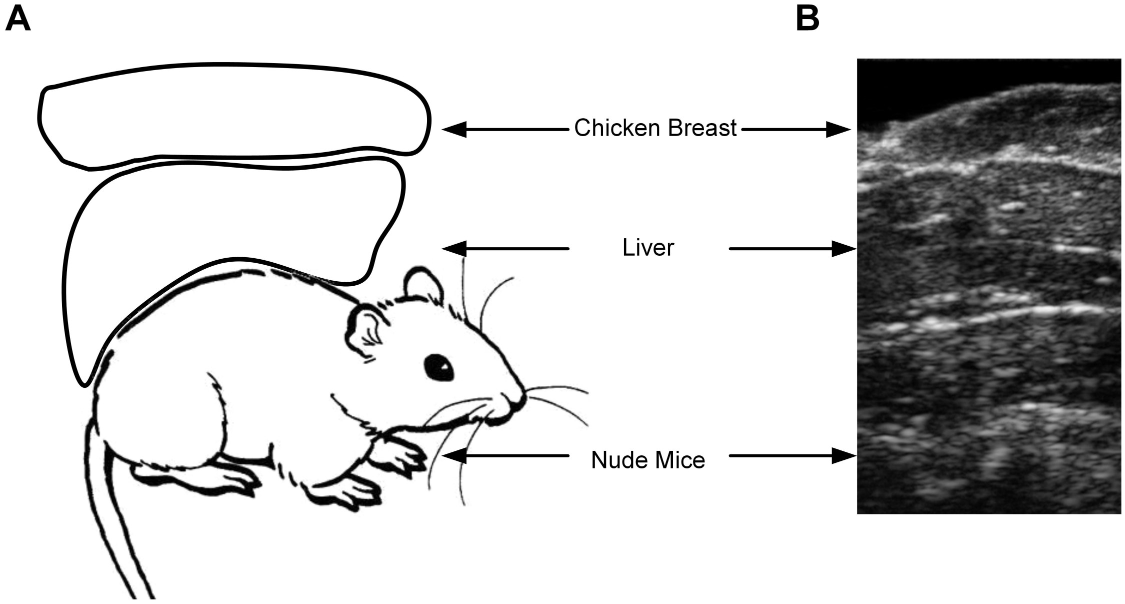

Figure S5. Structure (A) and validation (B) of the *in vivo* heterozygosity model.

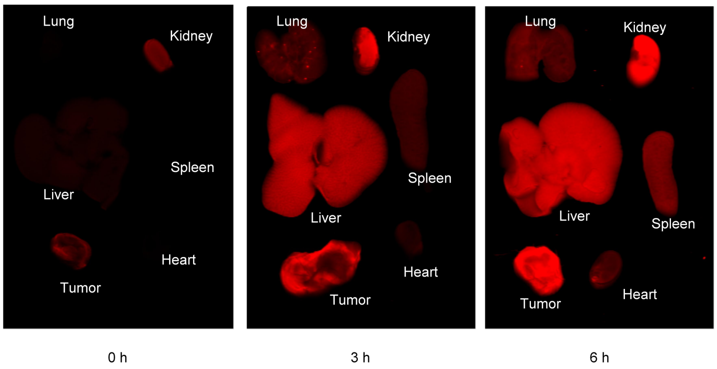

Figure S6 *Ex vivo* fluorescence images of tumors and major organs.

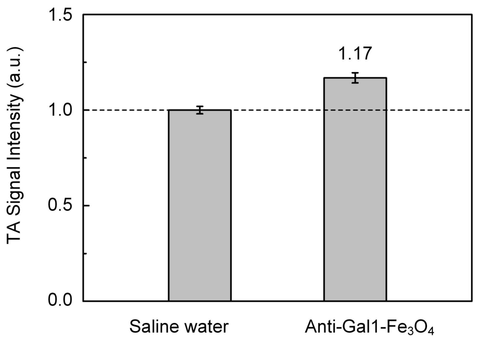

Figure S7 MTAI signal intensity of saline water and anti-Gal1-Fe_3O_4 nanoparticles with 1 mg/mL. The error bars represent triplicate samples and measurements.

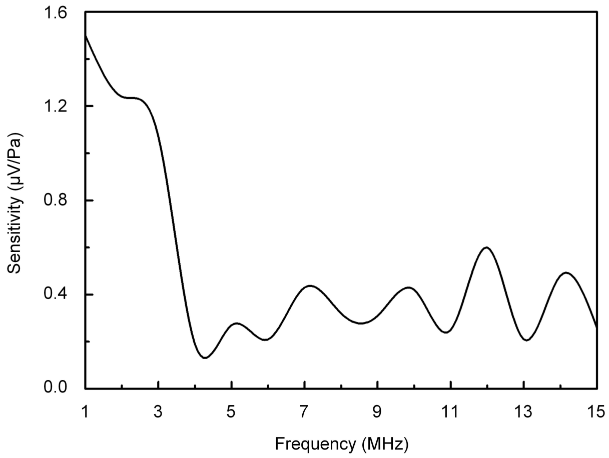

Figure S8 µV/Pa vs frequency plot of probe sensitivity.

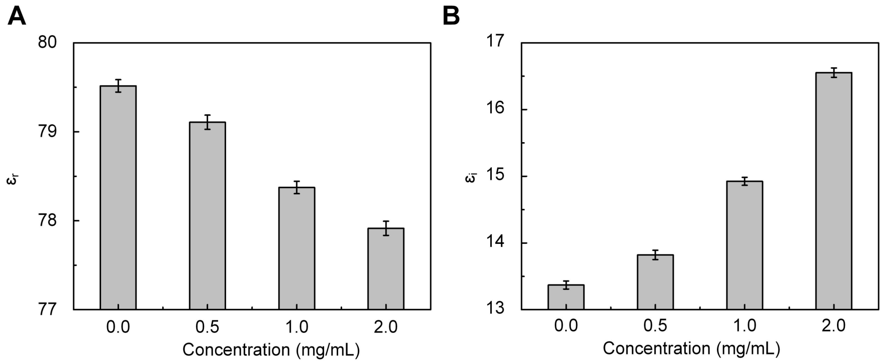

Figure S9. Permittivity real part (ε_r), imaginary part (ε_i) of anti-Gal1-Fe_3O_4 solution with different concentrations at 3GHz.

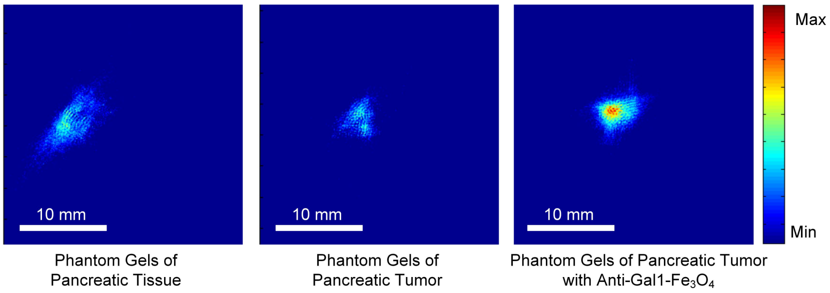

Figure S10. MTAI of different phantom gels.

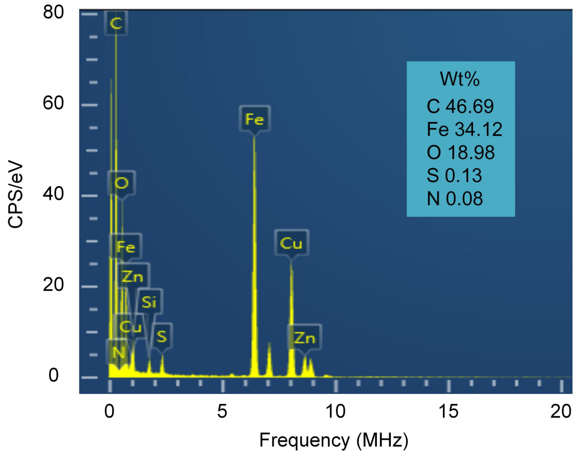

Figure S11. Energy spectrum distribution of anti-Gal1-Fe_3O_4.
